# Healthcare workers’ acceptability of influenza vaccination nudges: Evaluation of a real-world intervention

**DOI:** 10.1016/j.pmedr.2022.101910

**Published:** 2022-07-20

**Authors:** Rachelle de Vries, Mariëtte van den Hoven, Denise de Ridder, Marcel Verweij, Emely de Vet

**Affiliations:** a Chair Group Consumption & Healthy Lifestyles, Wageningen University & Research, the Netherlands; b Amsterdam University Medical Center, the Netherlands; c Department of Social, Health and Organizational Psychology, Utrecht University, the Netherlands; d Chair Group Philosophy, Wageningen University & Research, the Netherlands

**Keywords:** Influenza vaccination, Healthcare workers, Nudge acceptability, Vaccination status

## Abstract

Nudges have been proposed as an effective tool to stimulate influenza vaccination uptake in healthcare workers. However, the success of such nudges in practice is heavily reliant on their acceptance by the intended healthcare worker population, which has not been thoroughly examined to date. This study investigated healthcare workers’ acceptability of diverse influenza vaccination nudges implemented in a real-world vaccination campaign and explored the relationship between nudge acceptability and vaccination uptake. A cross-sectional study was conducted among 244 Dutch hospital employees, following a hospital-wide influenza vaccination nudging intervention. A survey assessed healthcare workers’ perceived acceptability of ten distinct influenza vaccination nudges, along with their vaccination status and relevant covariates (e.g., general perceptions regarding influenza vaccination of healthcare workers). Influenza vaccination nudges in general were deemed acceptable, with reward-based nudges being the least accepted, while digital vaccination forms, a mobile vaccination post, peer vaccination, and digital vaccination reminders were most appreciated. A higher overall acceptance of these nudges was associated with a greater likelihood of being vaccinated, particularly in healthcare workers with favorable perceptions of influenza vaccination usefulness. Our findings suggest that influenza vaccination nudges are an accepted means to systematically promote immunization of healthcare workers, and thus present a viable strategy for public health policies aimed at this group.

## 1 Introduction

Influenza is a recurring threat to public health systems worldwide, with recent figures estimating up to 650 000 annual cases of influenza-associated mortality globally (World Health Organization, 2019). Healthcare workers represent a key population to vaccinate for preventing the (direct and indirect) spread of infection in healthcare settings, especially to vulnerable patient groups (World Health Organization, 2019). Despite the demonstrated benefits of influenza vaccination, vaccination coverage of European healthcare workers rarely exceeds 30 % and remains well below recommended levels of (at least) 75 % (European Centre for Disease Prevention and Control, 2018). This is particularly the case in the Netherlands, where immunization rates oscillate between 13 and 28 percent (RIVM, 2021).

The current state of evidence thus points to a growing need for novel strategies to promote influenza vaccination of healthcare workers. Nudges – subtle alterations to the environmental context or “choice architecture” an individual operates within – have been shown to be an effective tool in this regard (see Renosa *et al*., 2021 for a recent review). In addition, in contrast with existing policies such as vaccination mandates, influenza vaccination nudges can gently incentivize immunization without having to violate an individual’s pre-existing attitudes or personal preferences regarding obtaining an influenza vaccine (Dubov & Phung, 2015).

Importantly, the ability of a nudge to successfully steer desirable behavior in practice is heavily reliant on its acceptance by the intended public (Luszczynska et al., 2020). While nudges are theoretically underpinned to preserve an individual’s freedom of choice, some evidence suggests that such subtle interventions can still be interpreted as intrusive or manipulative by receiving parties (Hagman et al., 2015). Moreover, within the domain of healthcare worker immunization, the acceptability and resulting efficacy of related nudges may be further hindered by specific challenges that vaccination poses for this profession: Influenza vaccinations are typically initiated in a “top-down” manner by hierarchical relations (e.g., hospital management), and the principal beneficiaries of vaccination are other individuals such as high-risk patients (i.e., influenza vaccination of healthcare workers can be considered primarily “other-regarding”; Van den Hoven, 2021).

However, little research has explicitly assessed to what extent influenza vaccination nudges administered by an employer (i.e., hospital) are perceived as acceptable by employees (Reñosa et al., 2021). Additionally, among the available literature, investigations have typically exposed and measured healthcare worker responses to a limited scope of influenza vaccination nudges at any one time (e.g., solely vaccination reminders; Barbaroux et al., 2021). In this study, we investigated healthcare workers’ acceptability of diverse influenza vaccination nudges implemented during a real-world vaccination campaign, which roughly embodied the three main categories of choice architecture techniques (i.e., *decision information*, *decision structure*, and *decision assistance* interventions; Münscher et al., 2016). Furthermore, we examined factors associated with influenza vaccination nudge acceptability, as well as explored the relationship between nudge acceptability and vaccination uptake.

## 2 Methods

### 2.1 Transparency and openness

This study was approved by the Social Sciences Ethics Committee of Wageningen University (number: 09215846) and all participants provided digital informed consent. Data were analyzed using SPSS (version 25) and are available to access at https://osf.io/f8y5p, where the complete outline of measures, pre-registered research questions and hypotheses, and pre-registered model fitting procedures are also detailed.

### 2.2 Participants and procedure

A cross-sectional study was conducted among healthcare workers at a large university hospital in the Netherlands, following a hospital-wide influenza nudging intervention in the months of October to November 2017. Participants were 244 hospital employees (34 % Nurse; 22 % Paramedic; 9 % Medical Doctor; 35 % Other: e.g., administrative and research staff) from various medical departments, most of which had direct contact with patients (93 %) and were employed for 1 year or longer (90 %) at the time of investigation.

A link to an online survey was sent out to all hospital employees approximately one week after the nudging intervention ended. The survey encompassed a series of questions on their experiences and perceptions of the influenza vaccination campaign. Responses on vaccination status were not obligatory and with three missing responses, a slightly smaller but comparable sample of 241 healthcare workers was analyzed for this outcome.

### 2.3 Influenza vaccination campaign

Ten distinct influenza vaccination nudges were implemented by the hospital management during the campaign, such as making vaccination-relevant information salient (i.e., decision information nudges), altering vaccination options, efforts, or rewards (i.e., decision structure nudges), and providing vaccination reminders (i.e., decision assistance nudges) (Münscher et al., 2016; Table 1). Nudges were implemented approximately-three weeks leading up to and including the campaign’s vaccination phase, and all healthcare workers had the opportunity to get vaccinated during the period the survey was active.

**Table 1:** Nudges used in the hospital-wide influenza vaccination campaign and corresponding categories.

Influenza vaccination nudge (Number)	Nudging category^1^
Department information meetings regarding influenza vaccination (1)	Decision Information
Displaying daily influenza vaccination rates of each department on the intranet (2)	
Digital forms instead of paper when receiving vaccination (3)	Decision Structure
Mobile vaccination post: The presence of a mobile vaccination post within departments for healthcare workers to get vaccinated (4)	
Peer vaccination: The opportunity of healthcare workers to vaccinate each other (5)	
Rewarding the department with the highest vaccination rate with a trophy (6)	
Rewarding the department with the highest increase in vaccination rates, as well as most peer vaccinations, with a cake (7)	
Digital newsletters and intranet announcements to inform healthcare workers about influenza vaccination (8)	Decision Assistance
Announcing influenza vaccination schemes on posters, flyers, and hospital television screens (9)	
Arrows and banners to increase visibility of vaccination posts throughout hospital (10)	

1 Taken from Münscher et al., 2016.

### 2.4 Measures and analyses

#### 2.4.1 Primary outcome variables

The perceived acceptability of influenza vaccination nudges was rated on a five-point Likert Scale anchored from “Very Bad” to “Very Good”, similar to prior research (Cronbach’s α = 0.85; Reñosa et al., 2021). Furthermore, healthcare workers self-reported their vaccination status by indicating whether they had obtained an influenza vaccination during the campaign (i.e., *yes* versus *no*).

#### 2.4.2 Demographic and covariate data

As a proxy of one’s attitude towards influenza vaccination, information on a healthcare worker’s frequency of past influenza vaccinations (i.e., *every year*, *sometimes*, or *never*) was collected. An individual’s general perceptions regarding influenza vaccination of healthcare workers (i.e., Usefulness, Importance, and Acceptability) were also measured, using single five-point Likert items with corresponding anchors (e.g., “Very Useless” to “Very Useful”). Among those vaccinated, the primary motivation for obtaining an influenza vaccination was subsequently gauged (i.e., “*To prevent that patients get the flu*”, “*To prevent that I get the flu*”, “*I was not invited to get the flu shot*”, or “*Other*”). Finally, awareness (i.e., *yes* versus *no*) of each influenza vaccination nudge was recorded, in addition to demographic characteristics such as a healthcare worker’s functional role, medical department(s), and length of employment (i.e., *less than 1 year* versus *1 year or longer*).

#### 2.4.3 Statistical analyses

We analyzed nudge acceptability with a random intercept linear mixed effects model (LMM), which included *Influenza Vaccination Nudge* as a fixed effect, *Participant* as a random effect (covariance structure: Variance Components), *Healthcare worker Role*, *Medical Department*, *Length of Employment*, general perceptions of healthcare worker immunization (i.e., *Usefulness*, *Importance*, *Acceptability*), *Frequency of Past Influenza Vaccinations*, and *Nudge Awareness* as additional fixed predictors, and *Nudge Acceptability* ratings as the dependent variable. We further explored whether overall nudge acceptability covaried with vaccination status in a logistic regression analysis, with average *Nudge Acceptability* scores, *Frequency of Past Influenza Vaccinations*, and the rated *Usefulness* of healthcare worker immunization entered as predictors in a sequential fashion. These (conceptually-relevant) predictors were selected based on inter-variable correlations and likelihood tests between nested models (Babyak, 2004). Finally, we ran a one-sample *t*-test on average *Nudge Acceptability* ratings, as well as a Chi-square goodness-of-fit test on motivations for obtaining an influenza vaccination among vaccinated employees.

## 3 Results

### 3.1 General acceptance of influenza vaccination nudges by healthcare workers.

**Fig. 1:**
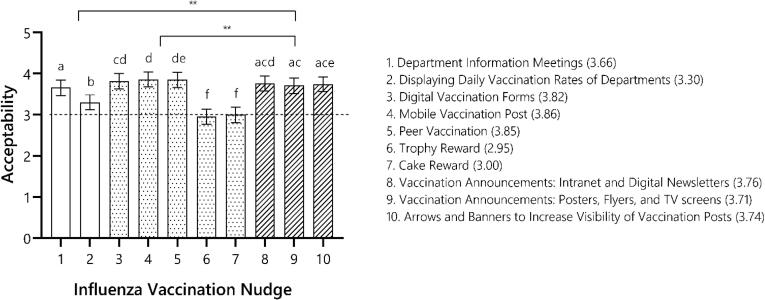
Average acceptability ratings across the ten influenza vaccination nudges (numbers 1 to 10 on the right) implemented during the hospital-wide vaccination campaign. Individual nudges with the same small letter did not significantly differ on perceived acceptability. At the category-level, decision assistance nudges (striped bars) were perceived as more acceptable overall, compared to both decision information (blank bars) and decision structure nudges (dotted bars). A double asterisk denotes a significant difference at *p* < 0.001 between nudge categories.

Influenza vaccination nudges were deemed acceptable overall, as average nudge acceptability ratings (3.81; standard deviation (SD) = 0.54) was significantly higher (i.e., more positive) than the neutral value of “3″, t(243) = 23.34, *p* < 0.001, *d* = 1.50. Systematic differences in perceived acceptability were found between influenza vaccination nudges, F(9,2175) = 70.67, *p* < 0.001, ηp^2^ = 0.23. Reward-based nudges (i.e., trophy and cake awards) were the least accepted, whereas digital vaccination forms, a mobile vaccination post, peer vaccination, and digital vaccination reminders were most appreciated by healthcare workers (Fig. 1). Moreover, individuals who reported being aware of a nudge, F(1,2300) = 113.19, *p* < 0.001, ηp^2^ = 0.05, as well as a higher acceptability of healthcare worker immunization, F(4,195) = 22.56, *p* < 0.001, ηp^2^ = 0.32, were generally more accepting of influenza vaccination nudges relative to their counterparts (Table S1 in the Supplemental Material). Nudge acceptability did not differ across healthcare worker roles or remaining predictors in the model (Table S1).

A supplementary category-based analysis yielded similar conclusions (Table S1), but also showed that reminder-based decision assistance nudges scored higher than either of the remaining nudge groups, F(2,2275) = 20.36, *p* <.001, ηp^2^ = 0.02 (*Decision Assistance* = 3.75, 95 % confidence interval (CI) = [3.57,3.92]; *Decision Information* = 3.49, 95 % CI = [3.31,3.67]; *Decision Structure* = 3.50, 95 % CI = [3.33,3.67]; both *p* < 0.001; Fig. 1).

### 3.2 Acceptability of influenza vaccination nudges is associated with vaccination status

154 employees (64 %) obtained an influenza vaccination during the hospital-wide campaign. Among vaccinated individuals, preventing that patients get the flu was the most frequently cited reason for getting vaccinated (60 %) – as opposed to more “self-regarding” motivations of preventing that healthcare workers themselves get the flu (33 %), χ^2^ (3) = 132.86, *p* < 0.001.

A higher overall acceptance of influenza vaccination nudges was associated with a greater likelihood of being vaccinated – both alone (odds ratio (OR) = 2.32, Wald statistic (1) = 9.54, *p* = 0.002) and after controlling for one’s frequency of past influenza vaccinations (OR = 2.10, Wald statistic = 5.46, *p* = 0.019; Table S2 in the Supplemental Material). However, this association was attenuated upon adjusting for an individual’s evaluated usefulness of healthcare worker immunization (OR = 1.33, Wald statistic = 0.51, *p* =.476), in which those that saw influenza vaccination as “very useful” were 5.92 times more likely to be vaccinated relative to those who assumed a neutral stance, Wald statistic = 6.64, *p* =.010 (Table S2).

## 4 Discussion

Diverse influenza vaccination nudges spanning a variety of choice architecture interventions were well-received by healthcare workers. Yet, we observed that an optimized selection of nudges includes those that decrease vaccination efforts (i.e., digital forms; peer vaccination; mobile vaccination post) and provide (digital) reminders, and excludes rewards. A potential explanation could be that healthcare workers appreciate strategies that largely resonate with their “other-regarding” perspective on influenza vaccination (Grant and Hofmann, 2011, Van den Hoven, 2021). Highly accepted nudges (e.g., peer vaccination) may better reinforce feelings of social collectiveness, whereas rewards may arguably undermine altruistic motives and seem patronizing. Alternatively, specific nudges (e.g., reminders) are useful for realizing pre-existing intentions towards vaccination and may therefore be preferred for their autonomy-preserving qualities (Münscher et al., 2016, Vugts et al., 2020). A more straightforward recommendation could then be to focus on these decision assistance nudges, as they consistently scored high on acceptability. Healthcare workers also tend to be more accepting of influenza vaccination nudges they are consciously aware of, and especially when they themselves independently approve of vaccination, which nicely complements extant work (Van Gestel et al., 2021, Reisch and Sunstein, 2016). These associations were consistent across nudging categories, suggesting that some acceptability correlates transcend differences in choice architecture design (e.g., nudges that appeal to “automatic” System 1 versus “deliberative” System 2 processing; Sunstein, 2016a).

While these findings are promising, observations also imply that the effectiveness of accepted nudges is subject to certain boundary conditions like a healthcare worker’s evaluated usefulness of influenza vaccination. The link between nudge acceptability and efficacy was particularly evident for individuals with favorable perceptions of influenza vaccination usefulness, likely because corresponding nudges were most embraced by those in the latter group (Van Gestel et al., 2021). As such, a healthcare worker’s “nudgeability” towards obtaining a vaccine appears to be moderated by one’s personal preferences regarding influenza vaccination (De Ridder et al., 2021). An ethically relevant implication of this is that influenza vaccination nudges seem to enable healthcare workers who are positively oriented towards immunization to act in line with their perceptions, whereas those with opposing beliefs are likely unaffected by these nudges (De Ridder et al., 2021) – which is well in line with the liberal philosophy endorsed by nudge theorists like Thaler and Sunstein (e.g., the “better off, as judged by themselves” standard; Sunstein, 2016b).

Our correlational study design and use of self-reports limit conclusions on the efficacy of nudging on overt vaccination behavior. Although, it is worth noting that experimental studies showed significant nudge-mediated increases in influenza vaccination rates (Milkman et al., 2021, Reñosa et al., 2021), which may be conditional upon certain situational (e.g., baseline vaccination levels; Barbaroux et al., 2021) or individual factors (e.g., perceived risk of infection; Ferguson & Gallagher, 2007). Furthermore, our sample consisted of majority vaccinated healthcare workers with predominantly favorable evaluations of influenza vaccination and direct patient contact, which questions the generalizability of our findings to the wider population of professionals. Finally, in addition to elucidating possible psychological mechanisms at play (e.g., autonomy feelings and nudge acceptability), future research should determine how viable these strategies are for other timely outbreaks such as the current COVID pandemic. Overlapping situational factors (i.e., “top-down” instruction from government bodies; strong “other-regarding” sentiments) indicate that COVID vaccination nudges may be similarly accepted by targeted parties. However, there could be a narrower window of opportunity for these nudges to work, given the more polarizing nature of the public COVID vaccination debate (Lazarus *et al*., 2021).

Taken together, our results suggest that influenza vaccination nudges are an accepted means to systematically promote immunization of healthcare workers and support the viability of such approaches in public health policies.

## CRediT authorship contribution statement

**Rachelle de Vries:** Conceptualization, Formal analysis, Visualization, Writing – original draft, Writing – review & editing. **Mariëtte van den Hoven:** Conceptualization, Funding acquisition, Methodology, Supervision, Writing – review & editing. **Denise de Ridder:** Conceptualization, Funding acquisition, Writing – review & editing. **Marcel Verweij:** Conceptualization, Funding acquisition, Methodology, Supervision, Writing – review & editing. **Emely de Vet:** Conceptualization, Funding acquisition, Methodology, Supervision, Writing – review & editing.

## Declaration of Competing Interest

The authors declare that they have no known competing financial interests or personal relationships that could have appeared to influence the work reported in this paper.
