# Supplementary material for: Healthcare workers’ acceptability of influenza vaccination nudges: Evaluation of a real-world intervention
Source: Prev Med Rep. 2022 Jul 20;29:101910. doi: 10.1016/j.pmedr.2022.101910 (PMC9326309; doi:10.1016/j.pmedr.2022.101910)
Supplement: Supplementary data 1 [file mmc1.docx]

**Supplemental Material for:**

**Healthcare Workers’ Acceptability of Influenza Vaccination Nudges: Evaluation of a Real-world Intervention**

**This file includes:**

Table S1

Table S2

**Table S1.**

Finalized linear mixed models (LMM) of influenza vaccination nudge acceptability (N=244).

|  | Nudge-based LMM ^a^ | B | 95% CI | | | | *p* | | ηp² | | |
| --- | --- | --- | --- | --- | --- | --- | --- | --- | --- | --- | --- |
|  | Intercept | 4.20 | 3.36 – 5.03 | | | | <.001* | | - | | |
|  | Influenza Vaccination Nudge | - | - | | | | <.001* | | 0.23 | | |
|  | Medical Department **^b^** | - | - | | | | .009* | | 0.27 | | |
|  | Length of Employment (Less than 1 year – 1 year or longer) | 0.29 | 0.10 – 0.48 | | | | .004* | | 0.04 | | |
|  | Nudge Awareness (Yes – No) **^c^** | 0.35 | 0.28 – 0.41 | | | | <.001* | | 0.05 | | |
|  | Acceptability Influenza Vaccination **^d^** | - | - | | | | | <.001* | 0.32 | | |
|  | Very Good – Very Bad | 1.09 | | 0.60 – 1.57 | | <.001* | | | | - |  |
|  | Very Good – Bad | 1.51 | | 1.10 – 1.92 | | <.001* | | | | - |  |
|  | Very Good – Neutral | 0.68 | | 0.50 – 0.87 | | <.001* | | | | - |  |
|  | Very Good – Good | 0.44 | | 0.28 – 0.61 | | <.001* | | | | - |  |
|  | **Category-based LMM ^a^** |  |  | |  | | |  |  | | |
|  | Intercept | 4.28 | 3.44 – 5.12 | | | | | <.001* | - | | |
|  | Nudging Category **^e^** | - | - | | | | | <.001* | 0.02 | | |
|  | Decision Assistance – Decision Information | 0.26 | 0.16 – 0.35 | | | | | <.001* | - | | |
|  | Decision Assistance – Decision Structure | 0.24 | 0.16 – 0.32 | | | | | <.001* | - | | |
|  | Decision Information – Decision Structure | -0.01 | -0.09 – 0.07 | | | | | .741 | - | | |
|  | Medical Department **^b^** | - | - | | | | | .010* | 0.27 | | |
|  | Length of Employment (Less than 1 year – 1 year or longer) | 0.29 | 0.10 – 0.49 | | | | | .004* | 0.04 | | |
|  | Nudge Awareness (% of Recognized Nudges in Nudging Category) **^c^** | 0.004 | 0.002 – 0.005 | | | | | <.001* | 0.01 | | |
|  | Acceptability Influenza Vaccination **^d^** | - | - | | | | | <.001* | 0.32 | | |
|  | Very Good – Very Bad | 1.09 | 0.60 – 1.58 | | | | | <.001* | - | | |
|  | Very Good – Bad | 1.52 | 1.10 – 1.93 | | | | | <.001* | - | | |
|  | Very Good – Neutral | 0.69 | 0.51 – 0.87 | | | | | <.001* | - | | |
|  | Very Good – Good | 0.45 | 0.29 – 0.61 | | | | | <.001* | - | | |

*Significant at α = 0.05

**^a^** Other (non-significant) predictors entered into the initial LMM: Healthcare worker role, perceived Usefulness of healthcare worker immunization, perceived Importance of healthcare worker immunization, and Frequency of past influenza vaccinations. Whether an individual had direct patient contact (*yes* versus *no*) also did not influence nudge acceptability in our sample.

**^b^** Many (N > 20) between-group differences were found. In the interest of brevity, we have omitted these results from the table.

**^c^** Did not differ across healthcare worker roles.

**^d^** Perceived acceptability of influenza vaccination of healthcare workers. In the interest of brevity, we only display group differences with the highest category (*Very Good*) as the reference.

**^e^** Exploratory interaction terms between Nudging Category and remaining predictors were not significant, indicating that similar factors accounted for acceptability across choice architecture techniques.

**Table S2.**

Exploring the relationship between influenza vaccination nudge acceptability and healthcare worker vaccination status in (nested) logistic regression models (N=241).

|  | Variable ^a^ | Exp (B) | 95% CI | Wald (df) | *p* | PAC ^b^ | -2LL^c^ | | |
| --- | --- | --- | --- | --- | --- | --- | --- | --- | --- |
| Model 1 | | | | | | | | |  |
|  | Constant | 0.07 | - | 6.38 (1) | .012* | 67.2 | 304.71 |  |  |
|  | Influenza Vaccination Nudge Acceptability | 2.32 | 1.36 – 3.96 | 9.54 (1) | .002* |  |  |  |  |
| Model 2 | | | | | | | | |  |
|  | Constant | 0.01 | - | 11.31 (1) | .001* | 79.7 | 209.71* |  |  |
|  | Influenza Vaccination Nudge Acceptability | 2.10 | 1.13 – 3.92 | 5.46 (1) | .019* |  |  |  |  |
|  | Frequency of Past Influenza Vaccinations | - | - | - | - |  |  |  |  |
|  | Every year – Never | 36.07 | 15.06 – 86.40 | 64.70 (1) | <.001* |  |  |  |  |
|  | Sometimes – Never | 4.18 | 1.74 – 10.00 | 10.28 (1) | .001* |  |  |  |  |
| Model 3 | | | | | | | | |  |
|  | Constant | 0.21 | - | 0.72 (1) | .398 | 82.6 | 185.50* |  |  |
|  | Influenza Vaccination Nudge Acceptability | 1.33 | 0.61 – 2.89 | 0.51 (1) | .476 |  |  |  |  |
|  | Frequency of Past Influenza Vaccinations | - | - | - | - |  |  |  |  |
|  | Every year – Never | 24.74 | 9.71 – 63.05 | 45.18 (1) | <.001* |  |  |  |  |
|  | Sometimes – Never | 4.17 | 1.62 – 10.74 | 8.77 (1) | .003* |  |  |  |  |
|  | Usefulness Influenza Vaccination ^d^ | - | - | - | - |  |  |  |  |
|  | Very Useful – Neutral | 5.92 | 1.54 – 22.7 | 6.64 (1) | .010* |  |  |  |  |

*Significant at α = 0.05 (two-tailed)

^a^ The selection of final variables was based on inter-variable correlations between predictors, as well as (-2 log likelihood) comparisons of model fit (cf. Babyak, 2004).

^b^ Percentage of sample cases correctly classified by model.

^c^ Model fit as assessed by the -2 log likelihood statistic. An asterisk indicates a significant improvement in model fit from the previous step.

^d^ Perceived usefulness of influenza vaccination of healthcare workers.
